# Patterns of follow up and survivorship care for people with colorectal cancer in new South Wales, Australia: a population-based survey

**DOI:** 10.1186/s12885-018-4297-6

**Published:** 2018-03-27

**Authors:** Jane M. Young, Ivana Durcinoska, Katie DeLoyde, Michael J. Solomon

**Affiliations:** 1 Surgical Outcomes Research Centre (SOuRCe), Sydney Local Health District and University of Sydney, Sydney, NSW Australia; 2 Institute of Academic Surgery, Royal Prince Alfred Hospital and University of Sydney, Sydney, NSW Australia; 3 Sydney School of Public Health, University of Sydney, Sydney, NSW Australia; 4 Centre for Oncology Education and Research Translation (CONCERT), Ingham Institute for Applied Medical Research & University of New South Wales, Liverpool, NSW Australia; 5 Discipline of Surgery, University of Sydney, Sydney, NSW Australia

**Keywords:** Cancer, Colorectal cancer, Survivorship, Surveillance, Disparities

## Abstract

**Background:**

The incidence and survival rates for colorectal cancer in Australia are among the highest in the world. With population growth and ageing there are increasing numbers of colorectal cancer survivors in the community, yet little is known of their ongoing follow up and survivorship care experiences. This study investigated patterns and predictors of follow up and survivorship care received and recommended for adults with colorectal cancer in New South Wales (NSW), Australia.

**Methods:**

Cross-sectional analysis within the NSW Bowel Cancer Care Survey, a population-based cohort of adults diagnosed with colorectal cancer between April 2012 and May 2013 in NSW. One year after diagnosis, participants completed a study specific questionnaire about their follow up and survivorship care experience and plans. Logistic regression was used to identify independent predictors of guideline-recommended care.

**Results:**

Of 1007 eligible people, 560 (56%) participated in the NSW Bowel Cancer Care Survey with 483 (86% of study participants, 48% of invited sample) completing the survivorship survey. Among these 483 participants, only 110 (23%, 95% Confidence Interval CI 19–27%) had received a written follow up plan, with this more common among migrants, non-urban dwellers and those with little experience of the health system. Of 379 (78%) people treated with curative intent, most were receiving ongoing colorectal cancer follow up from multiple providers with 28% (23–32%) attending three or more different doctors. However, less than half had received guideline-recommended follow-up colonoscopy (46%, CI 41–51%) or carcino-embryonic antigen assay (35%, CI 30–40%). Socio-economic advantage was associated with receipt of guideline-recommended care. While participants reported high interest in improving general health and lifestyle since their cancer diagnosis, few had received advice about screening for other cancers (24%, CI 19–28%) or assistance with lifestyle modification (30%, CI 26–34%). Less than half (47%, CI 43–52%) had discussed their family’s risk of cancer with a doctor since their diagnosis.

**Conclusions:**

Survivorship care was highly variable, with evident socioeconomic disparities and missed opportunities for health promotion.

**Electronic supplementary material:**

The online version of this article (10.1186/s12885-018-4297-6) contains supplementary material, which is available to authorized users.

## Background

Australia has one of the highest age-standardised incidence rates for colorectal cancer in the world, but also one of the highest 5-year relative survival rates (68%) for the disease [1, 2]. These factors, together with population growth and aging, are driving a rapid increase in the number of people in the community who have been previously treated for colorectal cancer (‘survivors’), challenging health services to provide equitable access to effective and cost effective survivorship and follow up care that meets patients’ needs.

Once patients have completed active treatment for incident colorectal cancer, further follow-up traditionally has focused on clinical surveillance to identify disease progression or recurrence. However, high-quality cancer survivorship care should also address other major issues that are important for patients’ health and well-being, including the management of any late or long-term physical or psychosocial sequelae of the cancer or cancer treatment. Furthermore, high quality survivorship care should encompass preventive interventions to reduce patients’ risk of developing new or recurrent colorectal cancer or other malignancies. Effective coordination of cancer-related and other health care services is also essential to ensure that patients’ care is streamlined and meets their individual needs [3].

For people with colorectal cancer, clinical practice guidelines for follow-up care have largely focused on recommendations for clinical surveillance with the aim of identifying recurrent disease or new tumours at an early enough stage for further potentially curative management. Such surveillance can include clinical examination, colonoscopy and carcino-embryonic antigen (CEA) assay as well as other diagnostic modalities. However, the clinical evidence underpinning recommendations for specific surveillance protocols in terms of timing, frequency and duration of follow up for specific patient sub-groups is somewhat limited, leading to varying guideline recommendations from different organisations internationally [4]. In Australia, national clinical practice guidelines recommend that people who have a colorectal cancer resection with curative intent and who are fit for further intervention have intensive follow up [5]. This should include a CEA assay every 6 months for 3 years and a surveillance colonoscopy at 12 months, then every 5 years if normal. Other aspects of survivorship care, including advice about lifestyle modification and screening for other cancers is at the clinician’s discretion. To improve survivorship care, peak cancer organisations in Australia endorse the Institute of Medicine’s recommendation that all patients who complete primary treatment for cancer should receive a care summary and survivorship care plan [6–8]. At present however, little is known about the delivery of survivorship care for people who have colorectal cancer in Australia.

Therefore this study was undertaken to describe the nature and patterns of follow up and survivorship care for a population-based sample of people with colorectal cancer in New South Wales (NSW), Australia’s most populous state. As previous studies have identified sociodemographic disparities in cancer care and outcomes [9–11], a further aim was to investigate variations in the receipt of care in accordance with national clinical practice guidelines by sociodemographic factors, specifically having private health insurance and geographical remoteness. It was hypothesised that patients without private health insurance and those living more remotely would be less likely to receive guideline-recommended care.

## Methods

### Study design

This study was a cross-sectional analysis within the NSW Bowel Cancer Care Survey, a population-based cohort study to assess the care coordination experiences and follow up care for adult patients with colorectal cancer [11].

### Procedure

A consecutive sample of patients notified to the NSW Cancer Registry between November 2012 and May 2013 were assessed for eligibility. Patients aged 18 years and older were eligible if they were diagnosed with incident primary colorectal cancer between October 2012 and March 2013, were aware of their diagnosis, were cognitively able to participate and were resident in NSW. Patients were considered ineligible if they had a life expectancy less than 6 months, had a previous colorectal malignancy or were long-term residents of a hospital or nursing home. Treating clinicians were notified of study eligibility criteria and were given the opportunity to exclude ineligible patients. Remaining patients were contacted by the research team by mail and asked to provide written consent. Consenting patients were asked to complete self-administered questionnaires at i) baseline (6–8 months post diagnosis) when they would have completed or nearly completed treatment and ii) follow up (12–14 months post diagnosis) when their follow up care arrangements had been established. A standardized reminder protocol was used to follow up non-responders. Additional study data were obtained from the NSW Cancer Registry. Findings from the baseline survey which focused on patients’ experience of care coordination during primary treatment have been reported in detail previously [11].

### Study instruments

The baseline questionnaire (6–8 months post-diagnosis) addressed patients’ socio-demographic characteristics (age, sex, ethnicity, education, employment, marital status, health insurance status (public or private), socioeconomic status, and residential remoteness) and clinical characteristics (comorbid conditions, health service utilization, presence of stoma, site and stage of disease and treatments received) and if they knew whether their case had been discussed at a multidisciplinary team (MDT) meeting. Additionally, in the baseline survey, patients were asked whether, prior to their cancer diagnosis, they had worked in the health system, had a close friend or relative working in the health system, had previous experience in the health system as a hospital patient or had previous experience with the health system through helping a friend or relative through their cancer treatment. Positive responses to these statements were categorised as ‘experience with the health system’. A positive response to the final response option for this question (‘Never had anything to do with the health system’) was categorised as ‘no experience’. Patients’ experience of cancer care coordination during their primary treatment for colorectal cancer was also assessed using the Cancer Care Coordination Questionnaire for Patients (CCCQ-P) [12]. This 20-item instrument, developed by our group, generates a care coordination score which ranges from 20 to 100 with higher scores indicating better experience. The instrument has robust psychometric properties with high internal consistency (Cronbachs’ α 0.88) and test re-test reliability (weighted Kappa > 0.40) for all items [12]. Geographical remoteness was assessed by the Accessibility/Remoteness Index of Australia (ARIA+) based on postcode of residence [13].

The follow up questionnaire (6 months after baseline) was designed specifically for this study (Additional file 1). This questionnaire asked about current health status and any cancer recurrence and then included questions addressing:

1. receipt of a written follow up or survivorship care plan
2. clinical follow up that had been undertaken since completing primary treatment (frequency and type of medical consultations and investigations including CEA assay and colonoscopy)
3. recommended future follow up (frequency and type of medical consultations, frequency and nature of future investigations including CEA assay and colonoscopy)
4. knowledge of symptoms to watch out for that could be due to bowel cancer
5. recall of discussion with a doctor about the risk of cancer for family members
6. level of interest in modifying lifestyle since cancer diagnosis
7. recall of advice from a health professional about screening for other cancers and lifestyle modification (smoking, alcohol, diet, physical activity, stress management).

### Sample size

The sample size for this study was determined by the sample size of the NSW Bowel Cancer Care Survey.

### Statistical analysis

Statistical analysis was conducted using SPSS 20.0 (IBM, USA) using two-tailed tests and a *p* <  0.05 significance level. Study participants and non-responders were compared with respect to age, sex, cancer site, stage of disease and residential remoteness (ARIA+) using chi square tests. Descriptive statistics were calculated for questionnaire responses. The mean and standard deviation (SD) are reported for all normally distributed data and the median and range for all non-normally distributed data. The primary outcomes were the proportion of patients who had:

- received a written follow up plan (all patients in cohort)

and for those treated with curative intent, who since completing treatment had:

- undergone a colonoscopy
- been recommended to have colonoscopy in the future
- undergone a CEA test
- been recommended to have CEA testing in the future.

Univariate associations between these outcomes and patients’ sociodemographic and clinical characteristics, baseline care coordination scores (analysed as two separate variables: on a continuous scale and dichotomized at the median) and experience with the health system were investigated using univariate logistic regression analysis. All variables with a *p* value of 0.2 or lower in univariate assessment were entered into a multivariable logistic regression base model. The model was then refined manually by elimination of the least significant potential predictor variable in a step-wise approach, until all remaining variables in the model were statistically significant [14]. Adjusted odds ratios (OR) and 95% confidence intervals (CIs) were calculated.

## Results

**Fig. 1:**
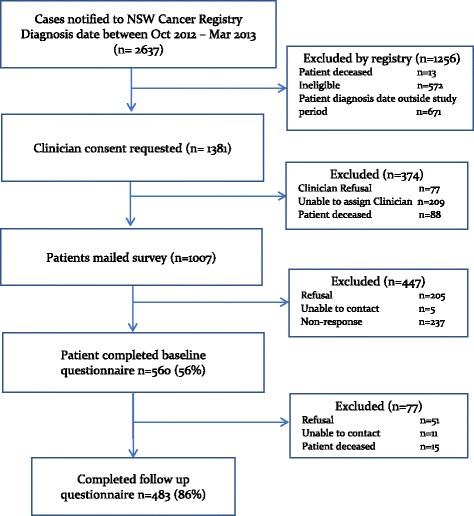
Study flow chart

Of the 1007 patients who were invited to participate, 560 completed the baseline questionnaire (response rate 56%), with results reported fully elsewhere [11]. The follow up questionnaire was completed by 483 (86% of baseline participants, 48% of invited sample) who comprise the study sample for the current analyses (Fig. 1).

As illustrated in Table 1, there were no statistically significant differences in sociodemographic or clinical characteristics of study participants and non-responders. Overall, the mean age of respondents was 68 years (SD = 12) and 60% were male (Table 1). The majority (*n* = 345, 71%) had colon cancer and 138 (29%) had rectal or rectosigmoid cancer (henceforth referred to as rectal cancer). The median cancer care coordination score was 75 (range 38–100) (Table 1). Overall, 379 (78%) respondents were treated with curative intent (279 with colon cancer and 100 with rectal cancer).

**Table 1:** Characteristics of participants (*n* = 483)

Characteristic		Participants (survey = 483)	Non-responders (*n* = 77)	*p*
		N (%)	N (%)	
Age	≤ 68 years	235 (49)	40 (52)	0.6
	> 68 years	248 (51)	37 (48)	
Sex	Male	289 (60)	46 (60)	0.9
	Female	194 (40)	31 (40)	
Cancer site	Colon	345 (71)	58 (75)	0.5
	Rectum	138 (29)	19 (25)	
Spread of disease	Local	148 (31)	22 (29)	0.9
	Regional	133 (28)	23 (30)	
	Distant	93 (19)	13 (17)	
	Missing	109 (23)	19 (25)	
Diagnosis	Symptoms	327 (69)		
	Routine Screening	149 (31)		
ARIA ^a^	Major city / Inner Regional	421 (87)	72 (94)	0.1
	Outer Regional / remote	62 (13)	5 (6)	
Language spoken at home	English	433 (91)		
Marital status	Single / divorced / widowed	145 (30)		
	Married / defacto / partner	338 (70)		
Education	Did not complete high school	92 (19)		
	Completed high school	170 (35)		
	Post-school education	219 (46)		
Employment	Full / part time work	110 (23)		
Private health insurance	Yes	265 (55)		
Lives alone	Yes	97 (20)		
Previous experience of health system	Yes	210 (44)		
Discussed at MDT^b^ meeting	Yes	163 (34)		
Self-reported health status	Excellent / very good	212 (44)		
	Good	197 (41)		
	Fair / poor	72 (15)		
Recurrence of colorectal cancer	Yes	33 (7)		
Cancer care coordination score	≤ 75	228 (50)		
	> 75	224 (50)		

^a^ARIA+ index – remoteness

^b^Multi-disciplinary team

### Receipt of written follow up care plan

Of the 483 participants, 299 (62%, 95% CI: 57–66%) indicated that they knew about symptoms they should be aware of that could be due to recurrent bowel cancer. However, only 110/483 (23%, 95% CI 19–27%) reported receiving a written follow up care plan. The proportion receiving a written plan was statistically significantly higher among those who spoke a language other than English at home (40% versus 21%, *p* = 0.005), those diagnosed through screening (30% versus 20%, *p* = 0.009), those living outside major cities (34% versus 21%, *p* = 0.03) and those with no previous experience of the health system as a patient (27% versus 18%, *p* = 0.02). These factors remained statistically significant in the multivariable model (Table 2). Although patients without private health insurance were also more likely to have received a written follow up plan in univariate assessment (28% versus 19%, p = 0.03), this factor was no longer statistically significant after adjustment for the other variables in the logistic regression model. There was no association between receiving a written follow up plan with marital status (*p* = 0.1), extent of disease at diagnosis (p = 0.1), seeing the same general practitioner at each visit (*p* = 0.2) or with experience of care coordination during primary treatment (OR 1.0, 95% CI 0.99–1.04, *p* = 0.11) and these variables were eliminated from the logistic regression model.

**Table 2:** Independent predictors of having received a written follow up care plan

Multivariable independent predictors	Adjusted *P* value	Adjusted OR	95% CI
Language spoken at home			
Not English	0.005	2.59	1.33–5.01
English		1.00	
Geographic location			
Outer regional/rural	0.03	1.96	1.05–3.65
City/inner regional		1.00	
Mode of diagnosis			
Screening	0.009	1.82	1.46–2.59
Symptoms		1.00	
Experience with health system			
No experience	0.02	1.86	1.48–2.69
Yes some experience		1.00	

*OR* Odds ratio, *CI* 95% confidence interval

### Patterns of follow up care among patients treated with curative intent

**Fig. 2:**
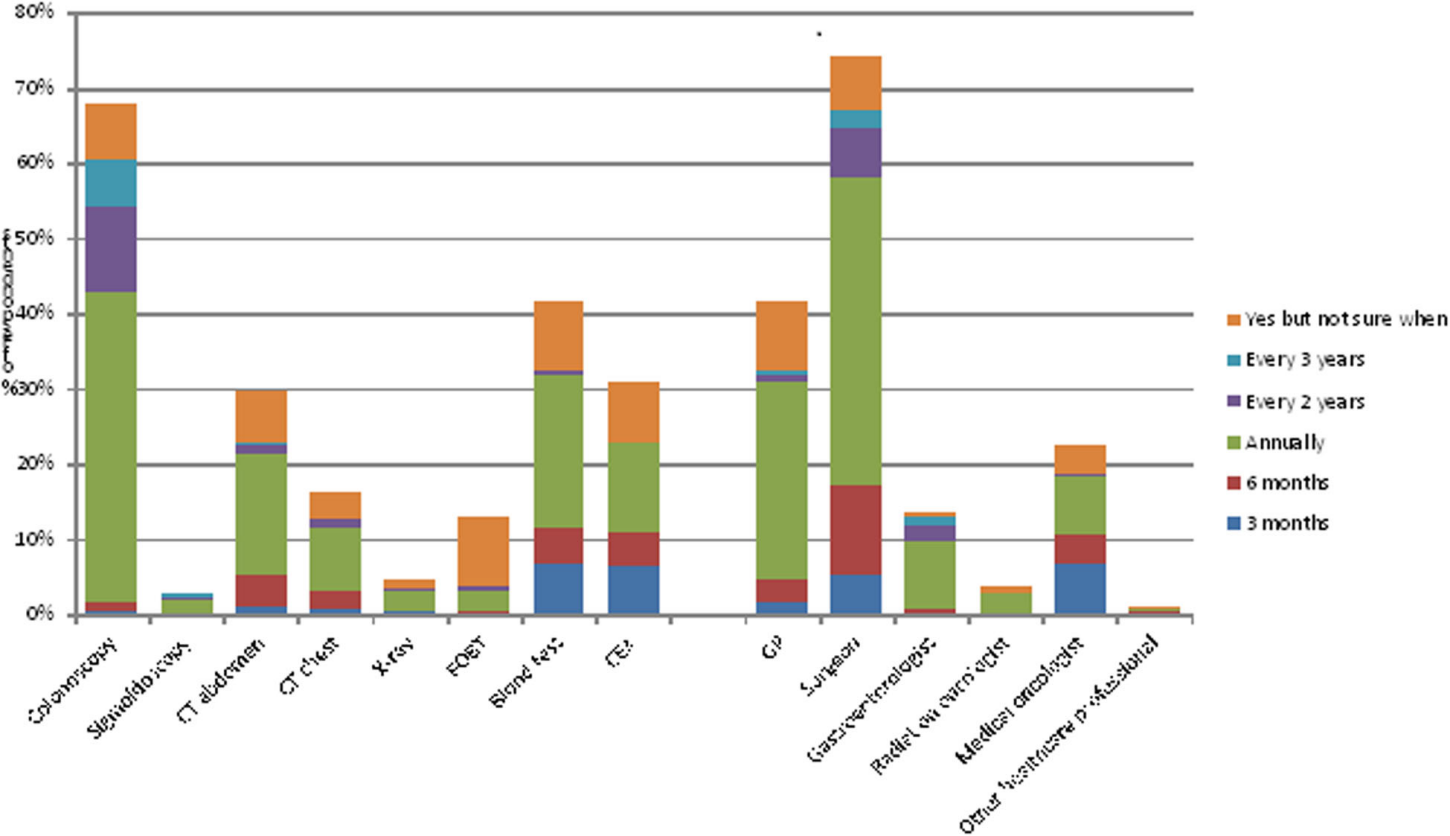
Percentage and timing of recommended future follow-up with clinical tests and health care professionals

Clinical follow up care received by the 379 patients treated with curative intent is summarised in Table 3. Ten patients (3%, 95% CI: 1–4%) reported not seeing any medical practitioner for ongoing colorectal follow up care. Most had been followed up by more than one type of doctor with 256/379 (68%, 95% CI: 63–72%) seeing two or more medical professionals and 105/379 (28%, 95% CI: 23–32%) seeing three or more (Table 3). The most common follow up investigations were routine blood testing followed by CT of the abdomen, colonoscopy and CEA testing, however the three latter investigations were reported by less than half of respondents (Table 3). Recommendations for ongoing follow up care and diagnostic testing were highly varied (Fig. 2).

**Table 3:** Clinical follow up of patients treated with curative intent (*n* = 379)

	n (%)
Number of medical practitioners seen for colorectal cancer follow up	
0	10 (3)
1	113 (30)
2	151 (40)
3	76 (20)
4	26 (7)
5	3 (< 1)
Types of medical practitioners seen for colorectal cancer follow up	
Surgeon	307 (81)
GP	261 (69)
Medical Oncologist	111 (29)
Gastroenterologist	45 (12)
Radiation Oncologist	22 (6)
Other Specialist	16 (4)
Diagnostic tests undertaken since completing colorectal cancer treatment	
Routine blood test	211 (56)
CT scan abdomen	176 (46)
Colonoscopy	173 (46)
CEA	133 (35)
CT scan chest	89 (24)
Chest X-ray	33 (9)
Sigmoidoscopy	14 (4)
FOBT	12 (3)
Barium enema	7 (2)

### Receipt of guideline-recommended intensive follow up for those treated with curative intent

#### a) Colonoscopy

Overall, 173/379 (46%, 95% CI: 41–51%) respondents treated with curative intent reported receiving a colonoscopy since completing primary treatment. This included 136/279 (49%) of those with colon cancer and 37/100 (37%) of those with rectal cancer (*p* = 0.04). Patients with private health insurance were also more likely to have had a colonoscopy (54% versus 37%, < 0.001) and these factors were found to be statistically significant, independent predictors in logistic regression modelling (Table 4).

**Table 4:** Independent predictors of guideline-concordant colorectal cancer intensive follow-up care

	Independent predictors	Adjusted P value	Adjusted odds ratio	95% confidence interval
Received follow up colonoscopy by 12 months	Private health insurance			
	Yes	< 0.001	1.63	1.38–2.10
	No		1.00	
	Cancer site			
	Colon	0.04	1.66	1.03–2.67
	Rectum		1.00	
Advised to have future colonoscopy	Private health insurance			
	Yes	0.007	1.82	1.08–3.06
	No		1.00	
	Written follow up care plan			
	Yes	< 0.001	3.41	1.64–7.07
	No		1.00	
	Self-reported health			
	Excellent/very good/good	0.03	2.33	1.16–4.70
	Fair/Poor		1.00	
	Mode of diagnosis			
	Screening / other	< 0.001	1.65	1.32–2.46
	Symptoms		1.00	
	Age			
	≤ 68 years	0.003	1.79	1.40–2.72
	> 68 years		1.00	
	Lives alone			
	No	< 0.001	1.60	1.30–2.34
	Yes		1.00	
	Stoma			
	No	0.03	1.55	1.28–2.18
	Yes		1.00	
Received follow up CEA test by 12 months	Age			
	≤ 68 years	0.001	1.55	1.32–1.97
	> 68 years		1.00	
	Level of education			
	College or university	0.024	1.68	1.09–2.59
	High school or less		1.00	
Advised to have future CEA testing	Employment status			
	Full or part time work	< 0.001	1.47	1.26–1.88
	Not in paid work		1.00	

Two-thirds (257/379, 68%: 95% CI: 63–72%) of respondents treated with curative intent reported being recommended to have ongoing colonoscopic surveillance as part of their future care (Fig. 2). This recommendation was more common among younger patients (77% versus 63%, *p* = 0.003), those diagnosed through routine screening (82% versus 64%, *p* <  0.001), those not living alone (75% versus 53%, *p* = < 0.001), those with better self-reported health (73% versus 56%, *p* = 0.03) and those without a stoma (74% versus 63%, *p* = 0.03). Furthermore, respondents with private health insurance (77% versus 64%, *p* = 0.007) and those who had been provided with a written follow up care plan (84% versus 62%, *p* < 0.001) were significantly more likely to have been advised to have ongoing colonoscopic surveillance (Table 4). These factors remained statistically significant in logistic regression modelling (Table 4).

#### b) CEA assay

Overall, 133/379 (35%, 95% CI: 30–40%) respondents treated with curative intent reported having had a CEA test following their treatment. This included 94/279 (34%) of patients with colon cancer and 39/100 (39%) of those with rectal cancer (*p* = 0.3). Although statistically significant univariate associations were found between having had a CEA test and a number of factors, including having private health insurance (41% versus 29%, *p* = 0.01), logistic regression analysis demonstrated that the only statistically significant, independent predictors were younger age and having a higher level of education (Table 4).

Less than a third of respondents (117/379 (31%, 95% CI: 26–36%)) reported being recommended to have ongoing CEA testing as part of their future care (Fig. 2). In univariate assessment, those with higher levels of education (39% versus 27%, *p* = 0.01) and those in paid employment (49% versus 27%, *p* = 0.01) were significantly associated with recalling advice to have ongoing CEA testing. However, after adjusting for employment status, level of education was no longer statistically significant and was eliminated from the final logistic regression model (Table 4). People with private health insurance were more likely to recall this advice to have ongoing CEA testing (37% versus 28%, *p* = 0.06) but this difference was not statistically significant in univariate or multivariable assessment.

### General health and lifestyle modification

Less than half (229/483, 47%, 95% CI: 43–52%) of respondents reported that a doctor had discussed their family’s risk of cancer following their own cancer diagnosis. Overall, only 114/483 (24%, 95% CI: 19–28%) participants recalled being advised to have regular screening tests for other cancers, including 28 (6%, 95% CI: 4–8%) advised to screen for skin cancer. Among women, 25/194 (13%, 95% CI: 8–18%) recalled advice to screen for breast cancer and 10 (5%, 95% CI: 2–9%) for cervical cancer whereas among men, 20/289 (7%, 95% CI: 4–10%) recalled advice to screen for prostate cancer.

The proportion of patients who reported having made greater effort to improve their lifestyle since their cancer diagnosis was highest for diet (226/483, 47%, 95% CI: 42–51%) and weight control (205/483, 42%, 95% CI: 38–47%) but more than one in four participants had made greater effort with each of the eight lifestyle factors in question (Table 5). Among the 306 who indicated they drank alcohol, 114 (37%, 95% CI: 32–41%) had mad more effort to reduce consumption since their cancer treatment and 31/70 (44%, CI 32–57%) smokers had made greater efforts to quit. In total, only 144/483 (30%, CI 26–34%) respondents reported receiving any assistance from a health professional with general health and lifestyle modifications since their diagnosis with colorectal cancer. Nearly one in five (87/483, 18%, 95% CI: 14–21%) reported that they would have like to have received greater assistance with general health and lifestyle modifications during this time period.

**Table 5:** Patients’ self-reported effort to improve lifestyle since cancer treatment

	Less effort	Same	More effort	Not applicable
Diet	7 (1)	202 (42)	226 (47)	6 (1)
Weight	11 (2)	216 (45)	205 (42)	8 (2)
Physical activity	29 (6)	225 (47)	170 (35)	7 (2)
Alcohol consumption	12 (2)	180 (37)	114 (24)	120 (25)
Smoking cessation	8 (2)	31 (6)	31 (6)	343 (71)
Sun protection	6 (1)	223 (46)	136 (28)	52 (11)
Stress	5 (1)	226 (47)	142 (29)	42 (9)
Sleep	10 (2)	267 (55)	123 (26)	20 (4)

## Discussion

This population-based survey of people previously treated for colorectal cancer found follow-up and survivorship care to be highly variable across NSW, with apparent socioeconomic differentials in the quality of care received. Despite multiple contacts with medical practitioners since completing primary treatment, the majority of participants in this study had not received guideline-recommended follow-up investigations and few recalled general health or lifestyle advice. Among those who had been treated with curative intent, over half (54%) had not received a follow-up colonoscopy and those without access to private health care were less likely to have done so. Furthermore, 53% of respondents had not had a discussion about the risk of colorectal cancer for their family since their own cancer diagnosis. With the burgeoning numbers of colorectal survivors in the community, strategies to standardise and better coordinate follow up and survivorship care are needed urgently to improve patient outcomes and to ensure equitable access to evidence-based approaches across all sectors of the population.

One of the key resources that has been advocated for by consumer groups is the provision of written care plans for patients to inform them about their future care pathway [6], with the Institute of Medicine strongly endorsing this approach [3]. Although fewer than one in four patients in this study recalled being given such a written plan, our findings suggest a form of positive discrimination. The specific patient groups who are known to have greatest difficulties with navigating the health system and accessing appropriate services, namely those living in regional and remote areas and those not speaking English as their first language as well as those with no experience of the health care system, were significantly more likely to have received a written care plan. This suggests the health system is capable of identifying and responding for patients perceived to be at increased risk, but that systems are not yet in place to ensure a standardised approach for all patients.

Intensive clinical follow-up for patients whose primary tumour was treated with curative intent was not widespread among survey respondents. There was an apparent paradox, with high levels of contact with medical practitioners yet substantial underuse of two of the major diagnostic modalities for identifying new or recurrent colorectal malignancy, namely colonoscopy and CEA testing. The low rates of uptake of these diagnostic modalities within the present study sample are broadly consistent with rates reported in other industrialised countries [15–18], highlighting that the delivery of guideline-recommended survivorship care is a challenge across different health systems. The reasons for this require further investigation so that the causes can be addressed. The relative weakness of the underlying scientific evidence base for effective approaches to colorectal cancer follow up is a major challenge, as it remains unclear what is optimal care. This is particularly an issue for surveillance colonoscopy, which is generally considered to have a role in the detection of metachronous disease rather than colorectal recurrence per se [19]. Inconsistent recommendations between different clinical practice guidelines, lack of agreed follow up care pathways and possible misperceptions among multiple health providers about who is responsible for coordinating ongoing diagnostic testing could also account for the apparently suboptimal care. Furthermore, patients’ general health or comorbidities, and their preferences for intensive follow up testing could explain some of the apparent low uptake of surveillance colonoscopy and CEA testing.

Differential financial and geographic access to health services is a plausible reason for the variations observed in this study. Access to health care is a multi-dimensional construct, comprising accessibility, availability, acceptability, affordability and adequacy as well as awareness of the service [20]. Patients with private health insurance, and thereby better access to private colonoscopy services, were significantly more likely to have received a post-treatment colonoscopy and to have been advised to have ongoing colonoscopic surveillance. Long waiting lists in the public sector and high out-of-pocket expenses impede access to colonoscopy services, requiring health policy solutions to improve equity. In comparison to colonoscopic surveillance however, the association between private health insurance and CEA testing was less strong but there were significant associations with other measures of socioeconomic status, namely education and employment levels. In addition to policy development to improve equity of access, strategies that support health professionals’ communication about the role of biomarkers such as CEA in disease surveillance, particularly for those with low health literacy, could be developed and tested to reduce disparities in this aspect of colorectal cancer survivorship care.

Although patients from regional and rural area have been found to have worse access to cancer services and poorer survival outcomes in other studies [21, 22], there was no evidence of geographical disparities in this study. Patients from regional and rural areas were in fact more likely to have received a written follow up care plan. Geographic location was not found to be associated with receipt of guideline recommended follow up care for those treated with curative intent. However this is in the context of sub-optimal survivorship care across the state. Vigilance is needed to ensure that any new strategies to improve the efficiency and effectiveness of follow up and survivorship cancer care do not disadvantage those living outside major cities.

The area of survivorship care that was not addressed for most patients was prevention. Few patients recalled any advice from a health professional about prevention or health promotion and only a minority had discussed relevant screening tests for other cancers. Despite this, patients expressed high levels of interest in lifestyle change to improve their health, with substantial proportions having made changes, particularly in diet and weight management, since their cancer diagnosis. The findings from this study provide endorsement from patients that these are important issues as many are keen to make lifestyle changes to improve their health and would welcome assistance from their doctors. The health system needs to develop effective strategies to ensure that all patients receive appropriate advice on prevention and health promotion, either from a specialist doctor, general practitioner or other member of their health care team.

### Limitations

Although a strength of this study was the use of a population-based registry to identify patients, the response rate of 56% among eligible patients invited to join the cohort leaves potential for selection bias. The representativeness of patients in the cohort has been reported previously [11]. However, among these participants, high rates of participation in the follow up survey (86%) were achieved. There was some variation in the timing of follow up survey in relation to completion of treatment, but all patients were more than a year post diagnosis and so should have had a post-treatment CEA assay and at least have a colonoscopy planned. The questionnaire was designed specifically for this study and warrants further validation to assess psychometric properties and reliability. Study findings are based on patient recall, which may not be completely accurate. During their treatment, patients receive large amounts of information from different sources at a stressful time in their lives, so may have difficulty recalling specific recommendations. However it is likely that patients would have good recall of having undergone specific tests such as colonoscopy. In terms of future surveillance testing and prevention however, even if the level of advice recalled by patients is an under-estimate of actual advice given, the results of this study show that the messages are not being recalled and better communication is needed. While our sample for this analysis was limited to NSW Bowel Cancer Care Survey participants, the achieved sample of 483 was sufficient to calculate 95% confidence intervals for the main study outcomes to ±5%. The study may have been underpowered to assess variations among small subgroups of patients. Finally, although the findings of this study are likely to be broadly generalizable across Australia and to jurisdictions with similar health systems, our results may not be generalizable to countries where primary and specialist health care is organised differently.

## Conclusions

This study found that colorectal cancer survivorship care was highly variable across NSW with less than half of patients receiving guideline-recommended surveillance colonoscopy or CEA assay a year after their diagnosis. There were evident socioeconomic disparities and missed opportunities for health promotion. Identifying effective policies and strategies to improve optimal survivorship care for all patients, regardless of their socioeconomic circumstances, must be a priority for health services to achieve the best possible outcomes for the rapidly-growing number of colorectal cancer survivors in the Australian community.

## Additional file

Additional file 1: NSW Bowel Cancer Care Follow-up Survey. (PDF 524 kb)
