# Supplementary material for: Patterns of follow up and survivorship care for people with colorectal cancer in new South Wales, Australia: a population-based survey
Source: BMC Cancer. 2018 Mar 27;18:339. doi: 10.1186/s12885-018-4297-6 (PMC5869767; doi:10.1186/s12885-018-4297-6)
Supplement: Supplementary file 1 — NSW Bowel Cancer Care Follow-up Survey. (PDF 524 kb) [file 12885_2018_4297_MOESM1_ESM.pdf]

The CONNECT Program
Research to improve care co-ordination and patient outcomes

THE UNIVERSITY OF SYDNEY

SOuRCe
Surgical Outcomes Research Centre

NSW Bowel Cancer Care
Follow-up Survey

This is the follow-up questionnaire to the **NSW Bowel Cancer Care Survey** you completed 6 months ago. This questionnaire asks about your follow-up care and arrangements, and gives you an opportunity to share your bowel cancer experience to help improve future care for patients with bowel cancer.

Any questions or concerns? Please call us on 1800 210 841 or email: connect@sswahs.nsw.gov.au

Thank you for your ongoing participation in this study.

1. What is today's date?

☐☐ / ☐☐ / 2 0 ☐☐

Your Health and Recovery

2. In general, would you say your health is:

- ☐ Excellent
- ☐ Very good
- ☐ Good
- ☐ Fair
- ☐ Poor

3. At any time since you were diagnosed with cancer, did a doctor or other health professional tell you that your cancer had come back (that is, you had a recurrence)?

- ☐ Not sure
- ☐ No
- ☐ Yes **Where is it located?**
  - ☐ Bowel
  - ☐ Lungs
  - ☐ Liver
  - ☐ Other*(please specify):* ____________________________

Sometimes, a patient will be given a **written follow-up plan** that outlines the follow-up arrangements and tests that are planned for the future.

4. Have you been given a written follow-up plan?

- ☐ Yes
- ☐ No
- ☐ Not sure
- ☐ I have been told that I will receive a written follow-up plan after I complete my treatment

Your Follow-up Care

5. Since completing treatment for your bowel cancer, have you seen any of the following health professionals for routine follow-up?

Please tick all that apply

- ☐ GP
- ☐ Surgeon
- ☐ Gastroenterologist
- ☐ Medical oncologist
- ☐ Radiation oncologist
- ☐ Other specialist *(please specify):* ___________________________

6. Have you had any of the below tests since completing your treatment?

Please tick all that apply

- ☐ Barium Enema
- ☐ Colonoscopy
- ☐ Sigmoidoscopy
- ☐ CT scan of abdomen
- ☐ CT scan of chest
- ☐ Chest X-Ray
- ☐ Faecal Occult Blood Test (FOBT)
- ☐ Routine blood test (blood count, blood tests for liver and kidney function)
- ☐ CEA test (specific blood test for bowel cancer)

Thinking about the future, the following questions ask about the arrangements for your follow-up care over the next 3 years. If you are unsure, please answer to the best of your ability.

7. Have you been advised to see any Doctor for routine follow-up of your bowel cancer over the next three years? If yes, please indicate how frequently you have been advised to see them.

Please tick all that apply

		Annually	Every 2 years	Every 3 years	Other *(please specify):*
GP	☐ Yes → ☐ No ☐ Not sure	☐	☐	☐	________________
Surgeon	☐ Yes → ☐ No ☐ Not sure	☐	☐	☐	________________
Gastroenterologist	☐ Yes → ☐ No ☐ Not sure	☐	☐	☐	________________
Radiation oncologist	☐ Yes → ☐ No ☐ Not sure	☐	☐	☐	________________
Medical oncologist	☐ Yes → ☐ No ☐ Not sure	☐	☐	☐	________________
Other *(please specify):* ________________	☐ Yes → ☐ No ☐ Not sure	☐	☐	☐	________________

8. Have you been advised to have any of the following tests or investigations in the next <u>three years</u>?
Please tick all that apply

		Annually	Every 2 years	Every 3 years	Other *(please specify)*:
Colonoscopy	☐ Yes → ☐ No ☐ Not sure	☐	☐	☐	________________
Sigmoidoscopy	☐ Yes → ☐ No ☐ Not sure	☐	☐	☐	________________
CT scan of abdomen	☐ Yes → ☐ No ☐ Not sure	☐	☐	☐	________________
CT scan of chest	☐ Yes → ☐ No ☐ Not sure	☐	☐	☐	________________
Chest X-Ray	☐ Yes → ☐ No ☐ Not sure	☐	☐	☐	________________
Faecal Occult Blood Test (FOBT)	☐ Yes → ☐ No ☐ Not sure	☐	☐	☐	________________
Routine blood test (blood count, blood tests for liver and kidney function)	☐ Yes → ☐ No ☐ Not sure	☐	☐	☐	________________
CEA test (specific blood test for bowel cancer)	☐ Yes → ☐ No ☐ Not sure	☐	☐	☐	________________

9. Which of the following statements best describes your preference for your follow-up care?

Please tick the box that most applies

- ☐ I would have preferred more follow-up consultations with my Doctors
- ☐ I would have preferred more follow-up investigations and tests
- ☐ I am happy with the follow-up consultations and investigations I received
- ☐ I would have preferred fewer follow-up investigations and tests
- ☐ I would have preferred fewer follow-up consultations with my Doctors

10. How would you rate the care you received from your follow-up care Doctor?

1 2 3 4 5 6 7 8 9 10

Very poor *Excellent*

Looking after yourself

11. Since your diagnosis with cancer, have you been advised to have regular screening tests for other cancers?

Please tick all that apply

☐ No

☐ Yes → ☐ Bowel cancer

☐ Skin cancer

☐ Breast cancer

☐ Cervical cancer

☐ Prostate cancer

☐ Other*(please specify)*: ________________________

12. Since your diagnosis with cancer, has your doctor discussed the risk of cancer for other members of your family?

Please note most cancers are NOT genetic and only a small number of families have a greater risk of cancer because they carry a changed gene

☐ No

☐ Yes

13. Since completing your cancer treatment, do you know what symptoms or changes in your health you should look out for that could be due to bowel cancer?

☐ No

☐ Yes

14. Since completing your cancer treatment, how much focus have you put in with other aspects of your lifestyle?

Please tick any that apply

	Less Effort	Same	More Effort	Not Applicable
Healthy diet	☐	☐	☐	☐
Maintaining a healthy weight	☐	☐	☐	☐
Being physically active	☐	☐	☐	☐
Limiting alcohol consumption	☐	☐	☐	☐
Stop smoking	☐	☐	☐	☐
Sun protection	☐	☐	☐	☐
Managing stress or anxiety	☐	☐	☐	☐
Getting enough sleep	☐	☐	☐	☐
Pap tests and mammography (women only)	☐	☐	☐	☐

15. Since completing your cancer treatment, have you received assistance from a health professional (eg. doctor, nurse, dietician, counsellor) with any of the above?

☐ No

☐ Yes

If yes, please describe:

____

16. Since completing your cancer treatment, would you like to have received more assistance, advice or support about general health or lifestyle issues?

☐ No

☐ Yes

If yes, which aspects of your lifestyle would you have liked more help with?

____

17. Since your diagnosis with cancer, have you or your family members used any services or resources that support coping or emotional well-being?

Please tick all that apply

☐ No, I did not need this type of support

☐ No, I did not know about these services

☐ Yes → ☐ Saw a psychologist/counsellor

☐ Attended a cancer support group

☐ Used internet and computer resources (eg. websites, online communities, blogs & online support groups)

☐ Used a telephone support helpline (eg. Cancer Council helpline)

☐ Other*(please specify)*: ___________________________

18. Did you have any comments or experiences that you would like to share?

____

____

____

Thank you very much for your time
